# Attenuation of amyloid‐β‐induced mitochondrial dysfunction by active components of anthocyanins in HT22 neuronal cells

**DOI:** 10.1002/mco2.301

**Published:** 2023-06-19

**Authors:** Jing Li, Pan Wang, Ming‐Jie Hou, Bao Ting Zhu

**Affiliations:** ^1^ Shenzhen Key Laboratory of Steroid Drug Discovery and Development, School of Medicine The Chinese University of Hong Kong Shenzhen Guangdong China; ^2^ School of Life Sciences University of Science and Technology of China Hefei Anhui China

**Keywords:** Alzheimer's disease, anthocyanin components, β‐catenin/TCF signaling pathway, mitochondrial dysfunction, mitochondrial homeostasis, petunidin

## Abstract

Alzheimer's disease (AD) is a common form of neurodegenerative disease in the elderly. Amyloid‐*β* (A*β*)‐associated neurotoxicity is an important component of the neurodegenerative change in AD. Recent studies have revealed a beneficial effect of anthocyanins in improving learning and memory in AD animal models. Using cultured HT22 mouse hippocampal neuronal cells as an in vitro model, we examined in this study the protective effect of ten pure components of anthocyanins against A*β*_42_‐induced cytotoxicity and also investigated the mechanism of their protective effects. We found that treatment of HT22 cells with the pure components of anthocyanins dose‐dependently rescued A*β*_42_‐induced cytotoxicity, with slightly different potencies. Using petunidin as a representative compound, we found that it enhanced mitochondrial homeostasis and function in A*β*_42_‐treated HT22 cells. Mechanistically, petunidin facilitated *β*‐catenin nuclear translocation and enhanced the interaction between *β*‐catenin and TCF7, which subsequently upregulated mitochondrial homeostasis‐related protein Mfn2, thereby promoting restoration of mitochondrial homeostasis and function in A*β*_42_‐treated HT22 cells. Together, these results reveal that the pure components of anthocyanins have a strong protective effect in HT22 cells against A*β*_42_‐induced cytotoxicity by ameliorating mitochondrial homeostasis and function in a *β*‐catenin/TCF‐dependent manner.

## Graphical abstract

A*β*_42_ suppresses *β*‐catenin/TCF signaling, leading to reduced expression of mitochondrial homeostasis‐related protein in HT22 cells. Petunidin protects against A*β*_42_‐induced cytotoxicity by restoring mitochondrial homeostasis, which is partly mediated by upregulation of the mitochondrial homeostasis‐related protein Mfn2 via promoting *β*‐catenin nuclear translocation and its interaction with TCF7.

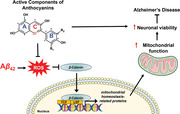

## 1 INTRODUCTION

The extracellular amyloid‐*β* (A*β*) deposition^1^, ^2^ and the intracellular neurofibrillary tangles (NFTs)^3^ are two pathological hallmarks of Alzheimer's disease (AD), which may contribute to neuronal death. Although there is still considerable controversy concerning the role of A*β* formation in AD pathogenesis,^4^, ^5^ increase in its formation is, nevertheless, a notable event and an important biomarker in AD development.^6^ A series of experimental evidence demonstrated that accumulation of A*β* could form A*β* oligomers and extracellular amyloid plaques, which are composed mostly of aggregated A*β*_42_ and A*β*_40_ peptides.^7^, ^8^ It is generally considered that A*β*_42_ plays an important role in the pathogenesis of AD^9^, ^10^ because A*β*_42_ fibril is a major component of the amyloid plaques and is also more neurotoxic than A*β*_40_.

A*β* accumulation can potentially lead to mitochondrial dysfunction,^11^, ^12^, ^13^ which has emerged as an important pathogenic change in AD. Mechanistically, A*β* may indirectly cause mitochondrial dysfunction through inducing free radical production and oxidative stress in hippocampal neurons of the AD brain.^14^ In addition, a series of findings have suggested that early accumulation of A*β* occurs in the import channels of the synaptic mitochondria,^15^ which have become an important pathogenic target of A*β* neurotoxicity.^16^, ^17^ In line with this suggestion, A*β* accumulation has been suggested to be associated with dysregulation of mitochondrial homeostasis and disruption of its electron transport chain complex.^18^ Experimentally, mitochondrial dysfunction often is reflected by findings showing impairment of the mitochondrial membrane potential (MMP), reduction of ATP production, and decrease of mitochondrial mass.^19^

Anthocyanins are natural water‐soluble flavonoid‐type compounds, and some of the identified bioactive components include cyanidin, malvidin, peonidin, delphinidin, and petunidin.^20^ Anthocyanins have many biological activities,^21^, ^22^ such as antioxidation,^21^ anti‐inflammation, anticancer activity and neuroprotection,^23^, ^24^ and are generally considered nontoxic. A recent study found that anthocyanins exerted a neuroprotective effect in neurodegenerative diseases by reducing oxidative stress and improving mitochondrial function.^24^ In an earlier study, it was observed that bilberry anthocyanins, which contained a mixture of natural compounds, could effectively reduce AD pathogenesis and improve learning and memory functions in an AD mouse model.^25^ However, it is not known whether some of the known components of anthocyanins also have a neuroprotective effect.

**FIGURE 1:**
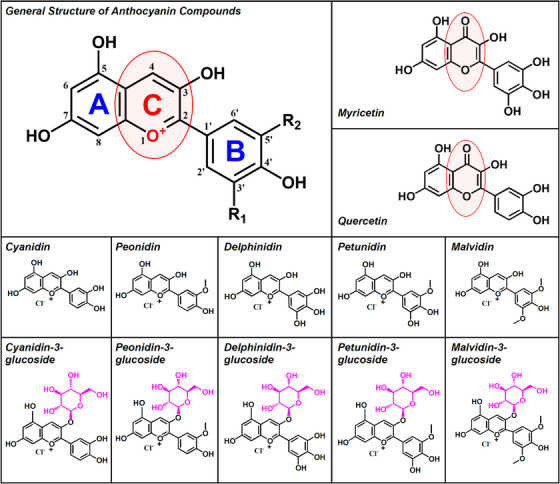
**Chemical structures of ten anthocyanins tested in this study**. The compounds include cyanidin, malvidin, peonidin, delphinidin, petunidin, and their respective glycosylated forms (i.e., cyaniding‐3‐glucoside, malvidin‐3‐glucoside, peonidin‐3‐glucoside, delphinidin‐3‐glucoside, petunidin‐3‐glucoside). For comparison, the structures of two commonly used flavonoids (myricetin and quercetin) are also shown. It is of note that the main difference between anthocyanins, myricetin and quercetin is in their *C*‐ring structures (highlighted by the red circles). While anthocyanins have a positive charge (O^+^) in their *C*‐rings, myricetin and quercetin do not have a similar positive charge in their respective *C*‐rings.

The present study aimed to investigate the neuroprotective effect of the pure components of anthocyanins and the underlying mechanism of their action. The immortalized HT22 mouse hippocampal neuronal cells in culture were used as an in vitro model and were treated with A*β*_42_ to induce cytotoxicity. Ten known pure components of anthocyanins, that is, cyanidin, malvidin, peonidin, delphinidin, and petunidin and their respective glycosides (structures shown in Figure 1) were selected for study. We found that these pure components of anthocyanins exerted protection against A*β*_42_‐induced cytotoxicity in HT22 cells, but with varying potencies. Mechanistic analysis using petunidin as a representative compound revealed that it improved cytotoxicity and promoted mitochondrial homeostasis through targeting the *β*‐catenin/TCF signaling pathway in A*β*_42_‐treated HT22 cells. These observations may form the basis for dietary intervention of A*β*‐associated AD pathology and its progression by using bioactive components contained in anthocyanins.

## 2 RESULTS

### 2.1 Pure components of anthocyanins alleviate Aβ_42_‐induced cytotoxicity

HT22 mouse hippocampal neuronal cells were treated with different concentrations (0.625–10 µM) of A*β*_42_ for 24 h. Based on the MTT assay, A*β*_42_ induced cytotoxicity in a dose‐dependent manner, with *IC*_50_ value of ∼5 μM (Figure S1A). Inspection of cellular gross morphology showed that the cells appeared to be less healthy after treatment with 5 or 10 μM A*β*_42_ for 24 h, although the cell density was not significantly altered (Figure S1B). Therefore, 5 μM A*β*_42_ was used in subsequent experiments to study the protective effect of selected anthocyanin components in HT22 cells and their mechanism of action.

Petunidin (one representative component of anthocyanins) at concentrations ranging from 2.5 to 40 μg/mL exhibited little cytotoxicity in HT22 cells (Figure S1C), but it restored the viability of A*β*_42_‐treated HT22 cells in a concentration‐dependent manner (Figure S1D). *N*‐Acetyl‐*L*‐cysteine (NAC), which is a well‐known antioxidant^26^ and was used as a positive control in this study for cytoprotection, also exerted a similarly strong protective effect against A*β*_42_‐induced cytotoxicity when present at a 40 mM concentration (Figure S1D). Notably, the cell numbers were not significant altered following treatment with A*β*_42_ or petunidin alone or both in combination for 24 h (Figure S1E). Importantly, petunidin at 5 μg/mL improved A*β*_42_‐induced change in the gross morphology of cells (Figure S1F), whereas no significant change in cell density was observed (Figure S1G). These results demonstrate that petunidin can ameliorate A*β*_42_‐induced cytotoxicity and morphological change in HT22 cells.

**FIGURE 2:**
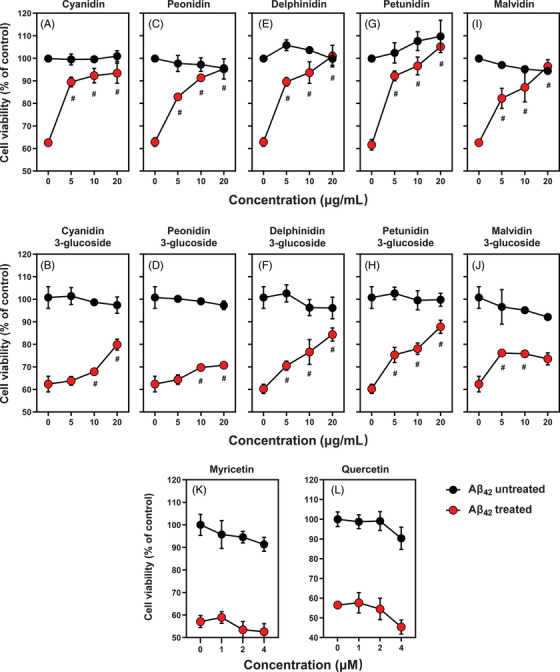
**Protective effect of the components of anthocyanins (cyanidin, malvidin, peonidin, delphinidin, petunidin, cyaniding‐3‐glucoside, malvidin‐3‐glucoside, peonidin‐3‐glucoside, delphinidin‐3‐glucoside, petunidin‐3‐glucoside) against A*β*_42_‐induced cytotoxicity in HT22 cells**. The concentration of A*β*_42_ used in this experiment was 5 μM, and the concentrations of each anthocyanin component were 5, 10, and 20 μg/mL. Quercetin and myricetin (at concentrations of 1, 2, and 4 μM) were also tested for comparison. Bars represent mean ± SD (*n* = 5). ^#^ *p* < 0.05 vs. A*β*_42_ treatment alone (i.e., without anthocyanins or flavonoids). Note that similar results (with slightly different concentration range) for petunidin has also been shown in **Figure** **S1**, and they are shown here again for convenience of comparison.

We also compared the effect of petunidin‐3‐glucoside, a glycosylated derivative of petunidin (structure shown in Figure 1) in A*β*_42_‐treated HT22 cells. Similar to petunidin, petunidin‐3‐glucoside also exerted a dose‐dependent protection against A*β*_42_‐induced cytotoxicity in HT22 cells, but apparently the potency of the glycosylated form was lower than the nonglycosylated form (Figure 2G and H). Similarly, we also tested the protective effect of other eight pure components of anthocyanins (cyanidin, malvidin, peonidin, delphinidin, and their respective glycosylated compounds) at concentrations from 5 to 20 μg/mL under the same experimental conditions (Figure 2A–F, I, and J). We found that each of the other four anthocyanin compounds (i.e., cyanidin, malvidin, peonidin, and delphinidin) exerted a similar dose‐dependent protection against A*β*_42_‐induced cytotoxicity in HT22 cells, although their potencies were slightly different. In comparison, the glycosylated compounds displayed a markedly reduced protective effect. It is apparent that the protective effect of anthocyanins is not dependent on the glycosylation, and in fact, glycosylation significantly reduces their neuroprotective effect in the in vitro cell culture model.

In this study, we also tested, for comparison, the protective effect of myricetin and quercetin (structures shown in Figure 1) against A*β*_42_‐induced cytotoxicity in HT22 cells under the same experimental conditions. Myricetin and quercetin share similar overall structures with some of the pure anthocyanin compounds tested in this study, but the formers lack a positive charge in their *C*‐ring structure. Interestingly, myricetin and quercetin did not have an appreciable protective effect against A*β*_42_‐induced cytotoxicity in HT22 cells (Figure 2K andL). This observation indicates that the protective effect of anthocyanins likely is associated with the unique positive charge of the anthocyanin compounds, which usually favors their preferential localization inside the mitochondria (discussed later).

Because petunidin was more potent than the other four anthocyanin compounds tested in this study, we thus chose to use petunidin as a representative anthocyanin compound to further study the protective mechanism against A*β*_42_‐induced cytotoxicity in cultured HT22 cells (data described below).

### 2.2 Petunidin attenuates Aβ_42_‐induced cellular ROS levels

**FIGURE 3:**
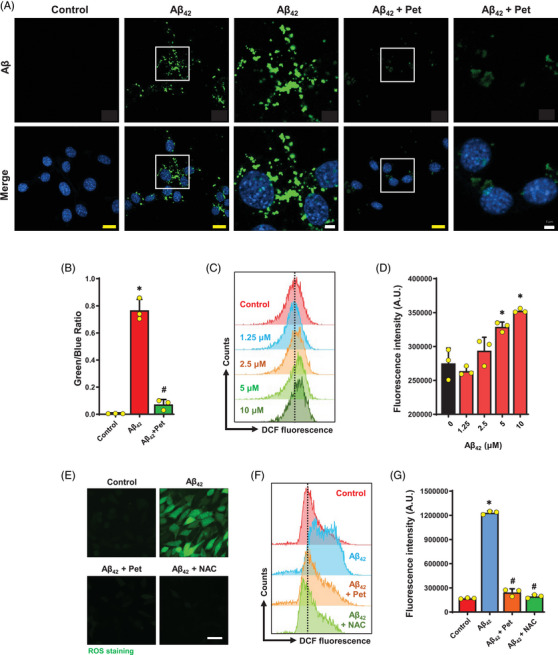
**Effect of petunidin on intracellular A*β* and ROS accumulation in A*β*_42_‐treated HT22 cells. (A)** Change in A*β* accumulation following treatment with 5 μM A*β*_42_ or A*β*_42_ + petunidin in combination for 24 h. Representative cellular images from different treatment groups and the magnification of selected cells in white box are shown (yellow scale bar = 20 μm, white scale bar = 5 μm). (**B)** Quantitative analysis of the image results in **A** (using the Image J Software) showing the green/blue fluorescence ratio, which represents the relative index for amyloid load. (**C, D)** Flow cytometry analysis of cellular ROS levels (labeled with 2′,7′‐dichlorodihydrofluorescein diacetate (DCFH‐DA)) following treatment with different concentrations of A*β*_42_ (1.25–10 μM) for 24 h. (**E)** Fluorescent microscopic images of cellular ROS levels (DCFH‐DA) following treatment with 5 μM A*β*_42_ alone or 5 μM A*β*_42_ + 5 μg/mL petunidin in combination or A*β*_42_ + 10 mM NAC in combination for 24 h. Scale bar = 50 μm. (**F, G)** Flow cytometry analysis of cellular ROS levels (labeled with DCFH‐DA) following the same treatment as in **E**. Bars represent mean ± SD (*n* = 3 or 5). *p < 0.05 vs. control group; ^#^ *p* < 0.05 vs. A*β*_42_‐treated group.

Accumulation of intracellular A*β* aggregates was observed following exposure to 5 μM A*β*_42_ for 24 h (Figure 3A and B). Petunidin and NAC each significantly reduced A*β*_42_ accumulation in A*β*_42_‐treated HT22 cells (Figures 3A and B and S2). A*β*_42_ was previously found to induce ROS accumulation, contributing to neuronal oxidative stress.^27^ In this study, we determined the ROS levels (staining with DCFH‐DA) in HT22 cells treated with A*β*_42_ or petunidin alone or in combination for 24 h. We found that treatment with A*β*_42_ increased fluorescence signal in a concentration‐dependent manner, with approximately a 30% increase over control when the cells were treated with 5 μM A*β*_42_ (Figure 3C and D).

Next, we determined whether A*β*_42_‐induced ROS accumulation could be reduced by treatment with petunidin. The result showed that after 24 h treatment, petunidin at 5 μg/mL effectively abrogated A*β*_42_‐induced ROS accumulation to similar levels as cells jointly treated with NAC (a ROS scavenger) (Figure 3E–G). Together, these results indicate that petunidin can protect HT22 cells against A*β*_42_‐induced oxidative stress.

### 2.3 Petunidin prevents Aβ_42_‐induced mitochondrial dysfunction and morphological alteration

**FIGURE 4:**
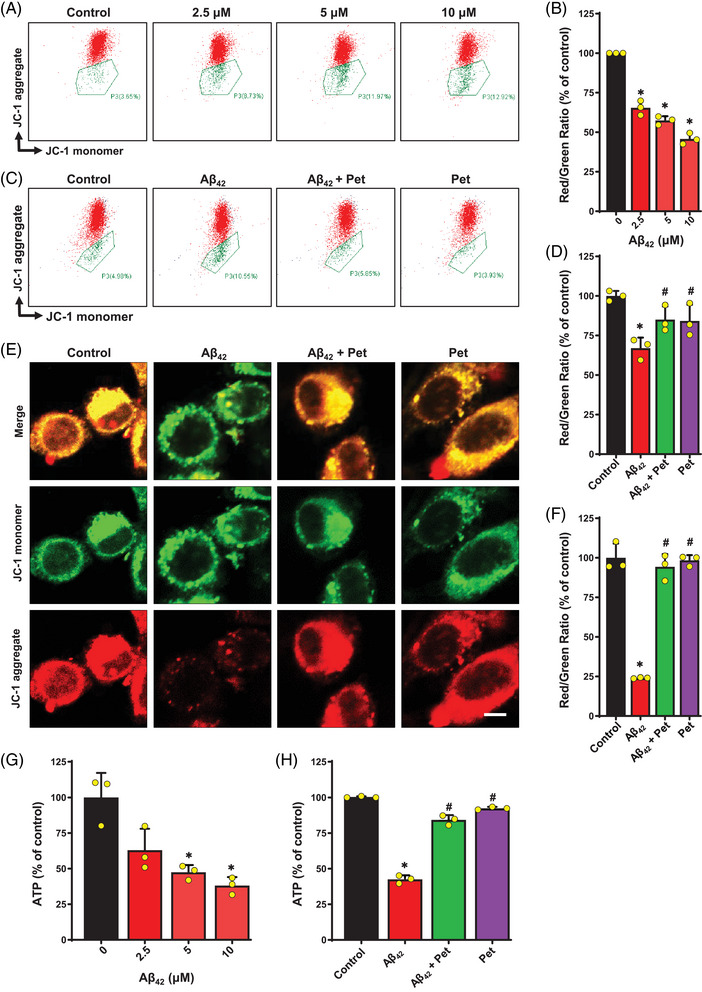
**Effect of petunidin on A*β*_42_‐induced mitochondrial dysfunction in HT22 cells. (A)** Mitochondrial membrane potential was analyzed using flow cytometry (labeled with JC‐1). The cells were treated with different concentrations of A*β*_42_ (2.5, 5 and 10 μM) for 24 h. (**B)** Quantitative analysis of the results in **A** showing the red/green fluorescence ratio, which represents the mitochondrial membrane potential. (**C)** Flow cytometry analysis of cells (labeled with JC‐1) following treatment with 5 μM A*β*_42_ or 5 μg/mL petunidin alone, or A*β*_42_ + petunidin in combination for 24 h. (**D)** Quantitative analysis of the results in **C** showing the red/green fluorescence ratio, which represents the mitochondrial membrane potential change. (**E)** Confocal microscopy analysis of cells (labeled with JC‐1) following the same treatment as in **C** (scale bar = 10 μm). A representative image from each group is shown. (**F)** Quantitative analysis of the image results in **E** (using Image J Software) showing the red/green fluorescence ratio, which represents the mitochondrial membrane potential change. (**G, H)** Change in cellular ATP levels following the same treatment as in **A** or **C**. Bars represent mean ± SD (*n* = 3). *p < 0.05 vs. control group; ^#^ *p* < 0.05 vs. A*β*_42_‐treated group.

Based on the above findings, we hypothesized that petunidin may ameliorate A*β*_42_‐induced cytotoxicity by targeting the mitochondria. Mitochondrial membrane potential and ATP levels were commonly used as indicators of mitochondrial function.^16^ Compared with the control group, treatment with A*β*_42_ increased the JC‐1 monomer green fluorescence intensity (Figure 4A and E) and reduced the JC‐1 aggregation (red fluorescence) to JC‐1 monomer (green fluorescence) ratio (Figure 4B), indicating a reduced mitochondrial membrane potential in A*β*_42_‐treated HT22 cells. Furthermore, the cellular ATP levels were decreased following treatment with A*β*_42_ (Figure 4G). Joint treatment of the cells with A*β*_42_ and petunidin decreased the green fluorescence intensity and restored A*β*_42_‐induced reductions in the red fluorescence/green fluorescence ratio (Figure 4C–F) and ATP levels (Figure 4H). These results indicate that petunidin likely protects the HT22 cells against A*β*_42_‐induced decreases in mitochondrial membrane potential and ATP levels, thereby restoring A*β*_42_‐induced mitochondrial dysfunction.

**FIGURE 5:**
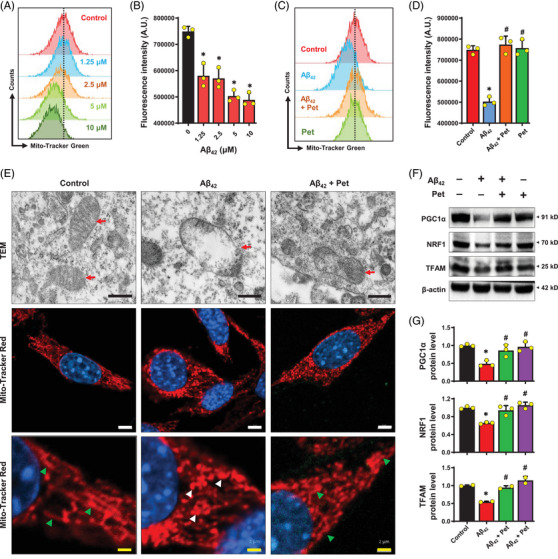
**Effect of petunidin on A*β*_42_‐induced change in mitochondrial morphology in HT22 cells. (A, B)** Flow cytometry analysis of cells (labeled with MitoTracker‐Green) following treatment with different concentrations of A*β*_42_ (2.5, 5 and 10 μM) for 24 h. (**C, D)** Flow cytometry analysis of cells (labeled with MitoTracker‐Green) following treatment with 5 μM A*β*_42_ or 5 μg/mL petunidin alone, or A*β*_42_ + petunidin in combination for 24 h. (**E)** Representative images of transmission electron microscopy (TEM) (scale bar = 500 nm) and MitoTracker‐Red staining (white scale bar = 5 μm, yellow scale bar = 2 μm) which showing the morphology of mitochondria. The cells were treated with 5 μM A*β*_42_ alone or A*β*_42_ + petunidin in combination for 24 h. Green arrows indicate branched healthy mitochondrial network. White arrows indicate spherical fragmented mitochondria. Red arrows point to the mitochondria. (**F, G)** Analysis of mitochondrial biogenesis‐related protein levels by Western blotting (**F**) and the quantitative analysis of the Western blotting results (**G**). Bars represent mean ± SD (*n* = 3). *p < 0.05 vs. control group; ^#^ *p* < 0.05 vs. A*β*_42_‐treated group.

Based on the above observations, next, we sought to assess the changes in mitochondrial morphology. Mito‐tracker green staining indicated that mitochondrial mass was decreased in A*β*_42_‐treated HT22 cells (Figure 5A and B). Furthermore, transmission electron microscopy analysis also suggested that the mitochondria morphology was altered in A*β*_42_‐treated HT22 cells with fewer mitochondrial cristae (Figure 5E). Notably, Mito‐tracker red staining showed that exposure of HT22 cells to A*β*_42_ induced mitochondrial fragmentation (Figure 5E), and joint treatment with petunidin significantly increased mitochondrial mass (Figure 5C and D), restored normal mitochondrial morphology, and attenuated mitochondrial fragmentation (Figure 5E) in A*β*_42_‐treated HT22 cells.

Next, we also examined the effect of A*β*_42_ and petunidin on cell levels of three mitochondrial biogenesis‐related proteins, namely, PGC1α, NRF1, and TFAM. We found that A*β*_42_ treatment significantly decreased the levels of PGC1α, NRF1, and TFAM in HT22 cells compared to the control (Figure 5F and G), and joint treatment with petunidin restored the levels of these three proteins in A*β*_42_‐treated cells (Figure 5F and G). These biochemical changes offer partial support for the subcellular morphological observations.

### 2.4 Petunidin activates β‐catenin/TCF signaling pathway in Aβ_42_‐treated HT22 cells

**FIGURE 6:**
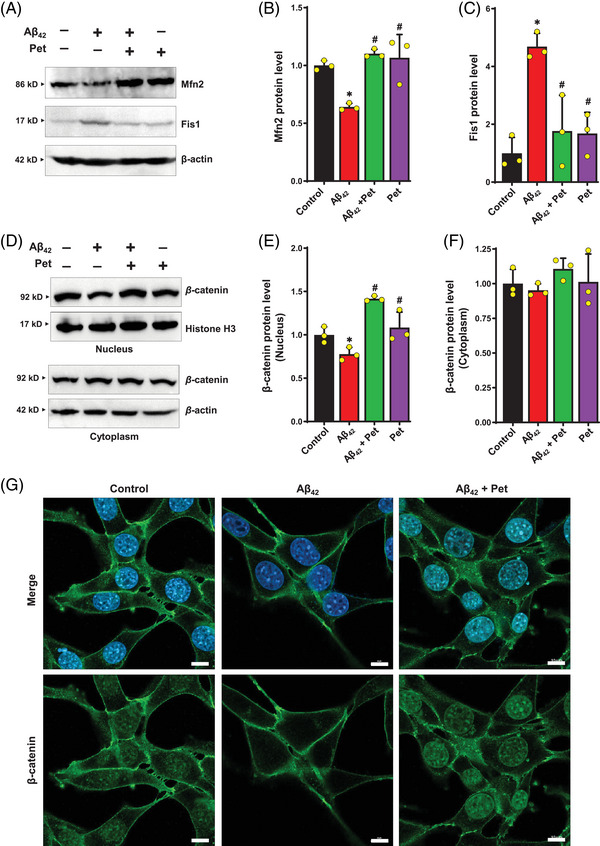
**Effect of petunidin on mitochondrial homeostasis‐related proteins and *β*‐catenin in A*β*_42_‐treated HT22 cells. (A‒C)** Analysis of mitochondrial homeostasis‐related protein levels by Western blotting (**A**) and the quantitative analysis of the Western blotting results (**B, C**). **D‒F**. Analysis of *β*‐catenin protein levels in the cytoplasm and nucleus by Western blotting (**D**) and the quantitative analysis of the Western blotting results (**E, F**). (**G)** Immunofluorescence of *β*‐catenin protein expression (scale bars = 10 μm). Bars represent mean ± SD (*n* = 3). *p < 0.05 vs. control group; ^#^ *p* < 0.05 vs. A*β*_42_‐treated group.

Previous studies showed that Mfn2, Fis1, and Opa1 genes encode mitochondrial membrane proteins and play a critical role in regulating mitochondrial homeostasis.^28^ Western blotting analysis of mitochondrial homeostasis‐related proteins suggested that A*β*_42_ treatment significantly decreased Mfn2 protein level but increased Fis1 protein level in HT22 cells compared to the control (Figure 6A–C). In addition, joint treatment of the cells with petunidin reversed these changes in A*β*_42_‐treated HT22 cells; that is, it significantly increased Mfn2 protein level but decreased Fis1 protein level (Figure 6A–C).

Next, we also investigated the possibility that petunidin might activate the *β*‐catenin/TCF signaling pathway. As shown in Figure 6D–G, A*β*_42_ treatment decreased the level of nuclear *β*‐catenin protein, with no significant change in the cytosolic *β*‐catenin protein level. Joint treatment of the cells with petunidin increased the level of nuclear *β*‐catenin protein compared to A*β*_42_‐treated group, with no significant change in the cytosolic *β*‐catenin protein level (Figure 6D–F). Immunofluorescence staining (Figure 6G) showed that petunidin significantly increased the level of *β*‐catenin in the nucleus, indicating that petunidin might increase the nuclear translocation of *β*‐catenin in A*β*_42_‐treated HT22 cells.

**FIGURE 7:**
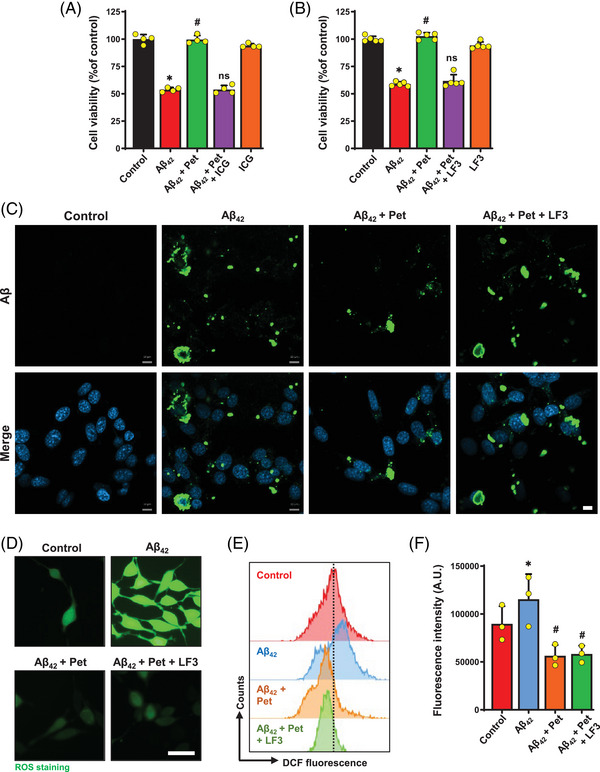
**ICG‐001 and LF3 abrogate the protective effect of petunidin against A*β*_42_‐induced cytotoxicity in HT22 cells**. Change in cell viability following treatment with 5 μM A*β*_42_ alone or 5 μM A*β*_42_ + 5 μg/mL petunidin or 5 μM A*β*_42_ + 5 μg/mL petunidin + 4 μM ICG‐001 (ICG) (**A**) or 5 μM A*β*_42_ + 5 μg/mL petunidin + 2.5 μM LF3 (**B**) in combination for 24 h. (**C)** Change in A*β* accumulation following treatment with 5 μM A*β*_42_ or A*β*_42_ + petunidin or A*β*_42_ + petunidin + LF3 in combination for 24 h. A representative image from each group is shown (scale bar = 10 μm). (**D)** Fluorescent microscopic images of cellular ROS levels (labeled with DCFH‐DA) following the same treatment as in **C** (scale bar = 50 μm). (**E, F)** Flow cytometry analysis of cellular ROS levels (labeled with DCFH‐DA) following the same treatment as in **C**. Bars represent mean ± SD (*n* = 3‒5). *p < 0.05 vs. control group; ^#^ *p* < 0.05 vs. A*β*_42_‐treated group; ns, not significantly different from A*β*_42_‐treated group.

Next, we further investigated whether the protective effect of petunidin against A*β*_42_‐induced cytotoxicity could be abrogated by joint treatment with ICG‐001 and LF3, which are known inhibitors of *β*‐catenin's transcriptional activity. The MTT assays showed that restoration of cell viability by petunidin in A*β*_42_‐treated HT22 cells was abrogated by joint treatment with ICG‐001 or LF3 (Figure 7A and B). Importantly, the reduction in cellular A*β* accumulation caused by 5 μg/mL petunidin in A*β*_42_‐treated HT22 cells was abrogated by joint treatment with LF3 (Figure 7C). However, the reduction of ROS by petunidin was not abrogated by LF3 (Figure 7D–F). These results indicate that while petunidin‐induced reduction of A*β*_42_ accumulation is mediated by activation of the *β*‐catenin/TCF signaling pathway, the reduction of ROS is not mediated by the same signaling pathway.

**FIGURE 8:**
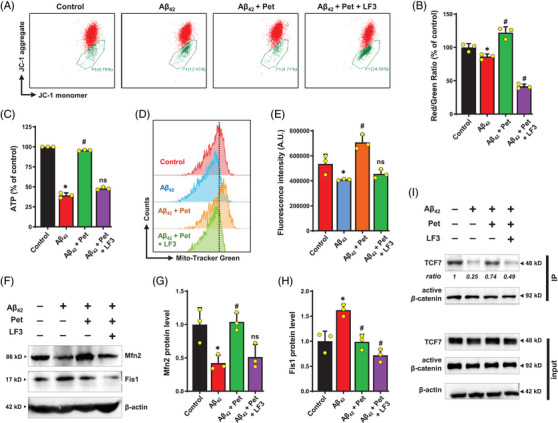
**LF3 abrogates the protective effect of petunidin against A*β*_42_‐induced mitochondrial dysfunction by suppressing *β*‐catenin/TCF interaction in HT22 cells. (A)** Change in mitochondrial membrane potential based on flow cytometry analysis (labeled with JC‐1) following treatment with 5 μM A*β*_42_ or A*β*_42_ + petunidin or A*β*_42_ + petunidin + LF3 in combination for 24 h. A representative data set is shown. (**B)** Quantitative analysis of the results in **A** showing the red/green fluorescence ratio, which represents the mitochondrial membrane potential change. (**C)** Change in cellular ATP levels following the same treatment as in **A**. (**D, E)** Flow cytometry analysis of cells (labeled with MitoTracker‐Green) following the same treatment as in **A**. (**F‒H)** Analysis of mitochondrial homeostasis‐related protein levels by Western blotting (**F**) and quantitative analysis of the Western blotting results (**G, H**). (**I)** Co‐IP analysis of *β*‐catenin and TCF7 following treatment with 5 μM A*β*_42_ alone or 5 μM A*β*_42_ + 5 μg/mL petunidin in combination or 5 μM A*β*_42_ + 5 μg/mL petunidin + 2.5 μM LF3 in combination for 24 h. Then cell lysates were immunoprecipitated with anti‐active *β*‐catenin antibody, and the immune‐precipitates were analyzed (Western blotting) for the active *β*‐catenin and TCF7 levels in the complex. Bars represent mean ± SD (*n* = 3). *p < 0.05 vs. control group; ^#^ *p* < 0.05 vs. A*β*_42_‐treated group; ns, not significantly different from A*β*_42_‐treated group.

Furthermore, we found that treatment with LF3 completely abolished the ability of petunidin to restore mitochondrial membrane potential (Figure 8A and B), cellular ATP content (Figure 8C), and mitochondrial mass (Figure 8D and E) in A*β*_42_‐treated HT22 cells. The results indicate that petunidin‐induced restoration of mitochondrial dysfunction likely is mostly mediated by activation of the *β*‐catenin/TCF signaling pathway.

Western blotting analysis showed that the small‐molecule inhibitor LF3 completely abolished the petunidin‐induced increase in Mfn2 protein level in A*β*_42_‐treated HT22 cells (Figure 8F and G) but did not alter the petunidin‐induced decrease in Fis1 protein level (Figure 8F and H). Furthermore, a co‐IP experiment was conducted to study the interaction of *β*‐catenin with TCF7. The total protein lysates of HT22 cells were immunoprecipitated with antibodies against active *β*‐catenin. We found that A*β*_42_ treatment suppressed the interaction of active *β*‐catenin with TCF7 in HT22 cells (Figure 8I); however, joint treatment of the cells with A*β*_42_ + petunidin enhanced the interaction of TCF7 with active *β*‐catenin, and the presence of LF3 abolished the enhanced interaction between the active *β*‐catenin and TCF7 in the cells (Figure 8I). Based on these observations, it is suggested that restoration of mitochondrial homeostasis by petunidin may be mediated through activation of the *β*‐catenin/TCF signaling pathway.

## 3 DISCUSSION

AD is the most common form of dementia in the elderly.^29^ Research that aims to identify new, safe, and protective agents against AD is in urgent need. Previous studies have reported that certain natural products may aid in delaying the progression of AD.^29^, ^30^, ^31^, ^32^ It was recently reported that bilberry anthocyanins reversed AD‐related cognitive dysfunction and reduced the hippocampal Tau neurofibrillary tangle number and A*β* levels in the APP/PSEN1 transgenic mice.^25^ In the present study, we found that several pure components of the bilberry anthocyanins effectively attenuated A*β*_42_‐induced cytotoxicity in cultured HT22 neuronal cells. Furthermore, the neuroprotective actions of petunidin were mediated through restoration of mitochondrial homeostasis and function via activation of the *β*‐catenin/TCF signaling pathway and upregulation of the mitochondrial homeostasis‐related protein Mfn2.

Mitochondrial function plays an important role in modulating neuronal survival.^19^ Mitochondrial dysfunction, which results in decreased mitochondrial membrane potential and ATP levels, drives the cognitive impairment in AD.^16^ Thus, attenuation of A*β*‐induced mitochondrial toxicity may represent an effective strategy for prevention or halting of AD perhaps at very early stages through dietary supplement‐mediated improvements of mitochondrial function. In this study, petunidin, a representative component of anthocyanins, was found to effectively attenuate A*β*_42_‐induced cytotoxicity, reduce A*β* accumulation, restore the mitochondrial morphology, and enhance mitochondrial biogenesis in cultured HT22 neuronal cells (Figures S1, S3, and S5). It is suggested that effective attenuation of A*β*_42_‐induced mitochondrial dysfunction, including decreased mitochondrial membrane potential and decreased ATP level in A*β*_42_‐treated HT22 cells (Figure 4), would be beneficial for preventing or halting the pathogenesis of AD.^17^ Furthermore, impairment of the mitochondrial function could decrease cellular ATP level, which likely would also hamper the degradation of the intracellular A*β*_42_.

Mitochondrial fusion‒fission dynamics is critical for maintaining mitochondrial homeostasis and neuronal survival and functions.^33^ Mitochondrial dynamics is controlled by several proteins, including Mfn1, Mfn2, and Opa1, which are responsible for mitochondrial fusion, and Drp1 and Fis1, which mediate mitochondrial fission.^34^ We found that petunidin significantly increased Mfn2 protein expression and decreased Fis1 protein expression in A*β*_42_‐treated HT22 cells (Figure 6). These observations indicate that petunidin may exert its cytoprotective effect in A*β*_42_‐treated HT22 cells through enhancing mitochondrial homeostasis to maintain normal mitochondrial biological functions.

Dysregulation of the Wnt signaling pathway has been suggested to be involved in AD pathology.^35^, ^36^ In this study, we found that petunidin significantly stimulated *β*‐catenin nuclear translocation (Figure 6) and promoted the *β*‐catenin/TCF interaction in A*β*_42_‐treated HT22 cells (Figure 8I). Two small‐molecule inhibitors (ICG‐001 and LF3) of the *β*‐catenin transcriptional activity effectively abrogated the protective effect of petunidin against A*β*_42_‐induced cytotoxicity in HT22 cells (Figure 7A–C). Interestingly, LF3 blocked petunidin‐induced upregulation of Mfn2 protein, but not downregulation of Fis1 protein (Figure 8F–H). Furthermore, we found that LF3 completely abolished the restorative effect of petunidin on cellular ATP level, mitochondrial membrane potential and mitochondrial mass in A*β*_42_‐treated HT22 cells (Figure 8A–E). However, petunidin‐induced reduction of cellular ROS levels in A*β*_42_‐treated HT22 cells was not affected by LF3 (Figure 7D–F). These results indicate that restoration of mitochondrial homeostasis and function, but not ROS, by petunidin is mediated by activation of the *β*‐catenin/TCF signaling pathway.

Structurally, the only difference between anthocyanins, myricetin, and quercetin is in their *C*‐ring structures (highlighted by the red circles in Figure 1). While anthocyanins have a positive charge (O^+^) in their *C*‐ring structures, myricetin and quercetin do not have a similar positive charge. Since earlier studies have demonstrated that many organic compounds with a positive charge, such as triphenylphosphonium (a lipophilic cation),^37^ rhodamine,^37^ methylene blue,^38^ and Mito‐TEMPOL,^39^ are favored to accumulate inside the mitochondria to exert their biological functions, it is, therefore, suggested that an important reason that the anthocyanin compounds are highly effective in protecting mitochondrial damage likely is due to the positive charge these compounds carry (Figure 1), which likely favors their preferential mitochondrial localization and protective action. By contrast, quercetin and myricetin, which are structurally similar to delphinidin and cyaniding but lack a positive charge in their structures, did not have any protective effect against A*β*_42_‐induced cytotoxicity under exactly the same experimental conditions used in this study (Figure 2K andL). Here it is also of note that our results with quercetin appeared to differ from those of a previous study^40^ reporting that quercetin exerted a protection in HT22 cells following exposure to A*β*_25‒35_. The discrepancy in these results might be related to the different A*β* fragments, different quercetin concentrations, and different treatment times used in the experiments.

Interestingly, the results of this study showed that the five nonglycosylated anthocyanin compounds have slightly different potencies for protection against A*β*_42_‐induced cytotoxicity. The rank order of potency is petunidin ≅ delphinidin ≅ cyanidin > peonidin ≅ malvidin (Figure 2). The difference in potencies of these components might be partly due to the different numbers and position of phenolic groups present. Petunidin, delphinidin, and cyanidin have two hydroxyl groups, but peonidin and malvidin only have one hydroxyl group. This might partly contribute to the observation that petunidin, delphinidin, and cyaniding have a slightly stronger protective effect than peonidin and malvidin. It is evident that the glycosylated anthocyanin compounds have a markedly weaker neuroprotective effect, which might be due to the following reasons: First, the glycosylated compounds have a lower permeability across the plasma membrane into the cells. Second, the metabolic conversion (hydrolysis) of glycosylated anthocyanins to the nonglycosylated active form is needed before the glycosylated compounds can exert their protective effect.

One of the potential limitations of our present study is the lack of in vivo data to demonstrate the effectiveness of pure anthocyanins in protection against Aβ_42_‐associated neuronal damage in animal models. The in vivo studies using relevant animal AD models might become more feasible in the future when sufficient amounts of pure anthocyanins become more affordable. Nevertheless, it should be noted that in our recent in vivo study using a transgenic AD animal model to test the effectiveness of a crude mixture of anthocyanins, it was observed that the crude mixture of anthocyanins has a strong protective effect against Aβ_42_‐associated neuronal damage in vivo (unpublished data). The in vivo protective effect with the crude mixture of anthocyanins offers partial support for the potential effectiveness of the pure anthocyanins in vivo.

In conclusion, the results of our present study demonstrated that pure anthocyanin compounds can effectively attenuate A*β*_42_‐induced cytotoxicity and restore mitochondrial homeostasis and function in cultured neuronal cells. Mechanistically, these compounds can promote the activation of the *β*‐catenin/TCF signaling pathways in A*β*_42_‐treated HT22 cells, which then enhances *β*‐catenin‒TCF interaction and results in upregulation of the mitochondrial homeostasis‐related protein Mfn2. Compared to other phenolics, the protective effect of anthocyanins likely is associated with the unique positive charge of the anthocyanin compounds. The findings of this study suggest that bioactive components of anthocyanins might have the potential to help halt the early stages of pathogenic progression of AD by ameliorating A*β*_42_‐induced hippocampal neuronal mitochondrial dysfunction and neurotoxicity. Future studies are needed to identify the direct cellular target(s) that might mediate the cytoprotective actions of anthocyanins.

## 4 MATERIALS AND METHODS

### 4.1 Chemicals and reagents

A*β*_42_ peptide ([H_2_N]‐DAEFRHDSGYEVHHQKLVFFAEDVGSNKGAIIGLMVGGVVIA‐[COOH], > 95% purity) was chemically synthesized by Shanghai Qiangyao Biotechnology (Shanghai, China). Cyanidin, malvidin, peonidin, delphinidin, petunidin (Pet), cyaniding‐3‐glucoside, malvidin‐3‐glucoside, peonidin‐3‐glucoside, delphinidin‐3‐glucoside, and petunidin‐3‐glucoside (>97% purity based on HPLC analysis; structures shown in Figure 1) were purchased from Shanghai Huicheng Biotech (Shanghai, China) and dissolved in double‐distilled water. Myricetin (≥96%, HPLC analysis) and quercetin (≥95%, HPLC analysis) were purchased from Sigma‐Aldrich (St. Louis, MO, USA). Dulbecco's Modified Eagle Medium (DMEM, 12800017) and fetal bovine serum (FBS) were purchased from GIBCO (GIBCO, Paisley, UK). Crystal violet staining solution (C0121), 2′,7′‐dichlorodihydrofluorescein diacetate (DCFH‐DA, S0033M), and JC‐1 working solution (C2003S) were purchased from Beyotime Biotechnology (Shanghai, China). Mito‐tracker red (M7512) was purchased from Thermo Fisher Scientific (Carlsbad, CA, USA). Mito‐tracker green (9074s) was purchased from Cell Signaling Technology (Danvers, MA, USA). *N*‐Acetyl‐*L*‐cysteine (NAC) was purchased from Sigma‐Aldrich (St. Louis, MO, USA) and dissolved in double‐distilled water. The ATP Assay Kit (S0026) was purchased from Beyotime Biotechnology (Shanghai, China). ICG‐001 (HY‐14428) was purchased from MedChemExpress (Monmouth Junction, NJ, USA). LF3 (S8474) was purchased from Selleck (Selleck Chemicals, Shanghai, China). Antibodies against Mfn2 (A12771), Fis1 (A5821), TCF7 (A20835), PGC1a (A12348), NRF1 (A5547), and TFAM (A13552) were purchased from Abclonal (Wuhan, China). Antibodies against *β*‐actin (3700S), A*β*_42_ (14974S), *β*‐catenin (8480S), and non‐phospho (active) *β*‐catenin (19807S) were purchased from Cell Signaling Technology (Danvers, MA, USA). HRP‐conjugated goat antirabbit IgG or rabbit anti‐mouse IgG was purchased from Proteintech (Wuhan, China).

### 4.2 Cell culture

HT22, an immortalized mouse hippocampal cell line subcloned from the HT‐4 cell line, is widely used as an in vitro model in the study of neurodegeneration.^41^ The HT22 mouse hippocampal neuronal cells were purchased from the Cell Bank of Shanghai Institute of Biological Science (Shanghai, China) and cultured in complete DMEM, supplemented with 10% FBS and 1% penicillin‐streptomycin medium. Cell culture dishes were kept at the 37°C atmosphere containing 5% CO_2_. Cells were passaged after reaching 60%–80% confluence. The passage of the cells used in experiments was limited to 10 times, and cells were authenticated by short tandem repeat profiling and routinely tested for mycoplasma contamination.

### 4.3 Cell viability assay

The HT22 cells were seeded in 96‐well plates at a density of 1500 cells/well and treated with different chemicals for 24 h. After treatment, the MTT assay was used to measure cell viability. The MTT solution (100 μL, at 0.5 mg/mL) was added to each well and cells were incubated for 3 h at 37°C, 5% CO_2_. After incubation, medium was removed and dimethylsulfoxide (DMSO, 100 μL) was added to each well to dissolve the MTT formazan. The absorbance was measured using a microplate reader (Biotek, Winooski, VT, USA) at 560 nm. The relative cell viability was compared to the vehicle control group.

### 4.4 Morphological analysis, cell counting, and crystal violet staining

Morphological changes of the cells in different treatment groups were visualized with a Nikon Eclipse Ti‐U inverted microscope (Nikon, Tokyo, Japan). The HT22 cells were seeded in 6‐well plates at a density of 5 × 10^4^ cells/well and treated with different chemicals for 24 h. After treatment, cells were trypsinized, and the cell number was determined using a hematocytometer as previously described.^42^ For crystal violet staining, cells were fixed with 1% glutaraldehyde for 15 min and stained with 50 μL of 0.5% (w/v) crystal violet solution (dissolved in 20% methanol and 80% deionized water) for 15 min at room temperature. Finally, the image was visualized and captured with a Nikon Eclipse Ti‐U inverted microscope.

### 4.5 Immunofluorescence staining

Immunofluorescence staining was performed to determine the A*β* and *β*‐catenin protein expression and the nuclear translocation of *β*‐catenin in the HT22 cells. Cells were cultured on cover slices for overnight. After that, cells were washed three times with PBS for 3 min each time and fixed with 4% paraformaldehyde in PBS for 15 min at room temperature. Cells were washed three times with PBS for 3 min each time and permeabilized with 0.1% Triton X‐100 in PBS for 10 min at room temperature. Cells were washed for three times with PBS for 3 min each time and blocked with 0.1% normal goat serum for 30 min at room temperature. Absorbent paper was used to absorb the blocking solution. Each slide was incubated with diluted primary antibody for overnight at 4°C in a humid box. Cells were washed for three times with PBST for 3 min each time and incubated with diluted Donkey anti‐Mouse IgG (H + L) Highly Cross‐Adsorbed Secondary Antibody (Alexa Fluor 488, A‐21202, Thermo Fisher Scientific) for 1 h at room temperature in the dark. Cells were washed for three times with PBST for 3 min each time and then counterstained with DAPI (20 μg/mL) for 5 min at room temperature in the dark. Finally, the image was visualized and captured (488/519 nm for the mouse Alexa Fluor 488‐conjugated antibody) under confocal laser scanning microscope (LSM 900; Carl Zeiss, Oberkochen, Germany).

### 4.6 Analysis of reactive oxygen species (ROS)

After treatment, cells were treated with 5 μM DCFH‐DA and incubated for 20 min at 37°C, 5% CO_2_. The DCFH‐DA‐treated cells were washed for three times with PBS. The fluorescence image was obtained with a Nikon Eclipse Ti‐U inverted microscope. The flow cytometry data were collected using the MoFlo XDP cell sorter (Beckman Coulter, Indianapolis, IN, USA) from FL1. A minimum of 10,000 cells was analyzed for each sample and data were processed with FlowJo (FlowJo, LLC, Ashland, OR, USA) software.

### 4.7 Mitochondrial membrane potential analysis

After treatment, cells were stained with 10 μg/mL JC‐1 working solution and incubated for 20 min at 37°C, 5% CO_2_. The JC‐1‐treated cells were washed for two times with JC‐1 dyeing buffer. Confocal imaging analysis was performed with a confocal laser scanning microscope at 490 nm (excitation) and 530 nm (emission) for JC‐1 monomers and 525 nm (excitation) and 590 nm (emission) for JC‐1 aggregates. JC‐1 fluorescence was quantified through flow cytometry analysis, in which red JC‐1 aggregate was measured at the FL2 channel and green JC‐1 monomer was measured at the FL1 channel. A minimum of 10,000 cells was analyzed for each sample with CytExpert (Beckman Coulter, Brea, USA) software.

### 4.8 ATP assay

ATP concentration was quantified by using ATP Assay Kit (Beyotime, S0026) according to the manufacturer's instructions.

### 4.9 Mito‐tracker staining

Cells were cultured on cover slides for overnight. After treatment, cells were stained with 200 nM Mito‐tracker red solution or 100 nM Mito‐tracker green solution and incubated for 20 min at 37°C, 5% CO_2_. Images were obtained by confocal laser scanning microscope at 579 nm (excitation) and 599 nm (emission) and were analyzed with Zen software (Carl Zeiss). The flow cytometry data were collected using the MoFlo XDP cell sorter from FL1. A minimum of 10,000 cells was analyzed for each sample with FlowJo software.

### 4.10 Transmission electron microscopy (TEM) analysis

After treatment, cells were carefully dissected and immersion‐fixed with 2.5% glutaraldehyde solution for 8 h at 4°C. The fixation solution was removed and cells were incubated with 1% osmic acid for 2 h at 4°C. Cells were dehydrated with gradient acetone solutions and soaked, embedded, and polymerized with Epon 812 epoxy resin. Finally, ultrathin section was prepared using an ultrathin slicer (0.5 μM, EM UC7, Leica, Wetzlar, Germany) and stained with 5% uranium dioxide‐acetate and 0.25% lead citrate for observation with transmission electron microscope (HT7700, Hitachi, Tokyo, Japan).

### 4.11 Western blot analysis

After treatment, HT22 cells were carefully collected at 3000 rpm for 3 min, washed in PBS for one time, and lysed using RIPA lysis buffer with protease inhibitors for 30 min on ice. The supernatant was collected for 8% or 10% SDS–PAGE analysis, and the proteins were then transferred onto PVDF membranes (Millipore, Lincoln Park, NJ, USA). PVDF membranes were blocked with 5% milk, washed with PBS, and incubated with primary antibodies for overnight at 4°C. After washing, the membranes were incubated with HRP‐conjugated secondary antibodies for 1 h at room temperature. Finally, the membranes were developed using Supersignal™ West Pico PLUS Chemiluminescent Substrate (ThermoFisher, Waltham, MA, USA). The band intensity of Western blot images was quantified with Image J Software.

### 4.12 Co‐immunoprecipitation analysis

For co‐immunoprecipitation (co‐IP), cell lysates were incubated with the antibodies against active *β*‐catenin (1 μg/100 μg total lysate) for 1 h at 4°C. The lysates were then incubated with the prewashed protein A/G agarose beads (20421, ThermoFisher) for 4 h at 4°C. After washing, the beads were mixed with 2✗ SDS‐loading buffer, boiled for 10 min at 100°C, and used for Western blot analysis.

### 4.13 Statistical analysis

Results shown are the mean ± SD from at least three measurements or independent experiments. Statistical analysis was performed with GraphPad Prism 9.0 software (GraphPad, San Diego, CA, USA). For multiple comparison analysis, one‐way ANOVA followed by Tukey's multiple comparison tests was performed.

## AUTHOR CONTRIBUTIONS

Jing Li: conceptualization, methodology, software, investigation, formal analysis, writing—original draft. Pan Wang: visualization, conceptualization, writing—review & editing. Ming‐Jie Hou: investigation. Bao Ting Zhu: conceptualization, funding acquisition, resources, supervision, writing—review & editing. All authors have read and approved the final version of the manuscript.

## CONFLICT OF INTEREST STATEMENT

There are no conflicts to declare.

## ETHICS STATEMENT

Not applicable.

## Supporting information

Supporting Information

## Data Availability

The data analyzed in this study are available from the corresponding author upon reasonable request.
