# Supplementary material for: Attenuation of amyloid‐β‐induced mitochondrial dysfunction by active components of anthocyanins in HT22 neuronal cells
Source: MedComm (2020). 2023 Jun 19;4(4):e301. doi: 10.1002/mco2.301 (PMC10279944; doi:10.1002/mco2.301)
Supplement: Supplementary file 1 — Supporting Information [file MCO2-4-e301-s001.pdf]

Attenuation of Amyloid-*β*-Induced Mitochondrial Dysfunction by Active Components of Anthocyanins in HT22 Neuronal Cells

Jing Li[1, 2], Pan Wang[1], Ming-Jie Hou[1] and Bao Ting Zhu[1, *]

[1]Shenzhen Key Laboratory of Steroid Drug Discovery and Development, School of Medicine, The Chinese University of Hong Kong, Shenzhen, Guangdong 518172, China

[2]University of Science and Technology of China, Hefei, Anhui, 230026, China

Running Title: **Neuroprotective Effect of Petunidin**

FOOTNOTES

*** Correspondence: BTZhu@CUHK.edu.cn**

SUPPLEMENTAL FIGURES

Figure S1. Effect of petunidin (Pet) against amyloid-$\beta_{1\text{-}42}$ (Aβ_{42})-induced cytotoxicity and cell morphology changes in HT22 cells.

A. To explore the toxicity of Aβ_{42} on HT22 cells, cells were treated with different concentrations of Aβ_{42} (0.625–10 μM) for 24 h, and cell viability was detected by MTT assay.

B. Change in gross cell morphology (a microscopic image of crystal violet-stained cells) following treatment with different concentrations of Aβ_{42} (2.5–10 μM). Scale bar = 25 μm. Red arrows indicate cells with abnormal morphology.

C. To explore the toxicity of petunidin on HT22 cells, cells were treated with different concentrations of petunidin (2.5–40 μg/mL) for 24 h, and cell viability was detected by MTT assay.

D. Protective effect of petunidin (1.25, 2.5 and 5 μg/mL) or *N*-acetyl-*L*-cysteine (NAC) (40 mM) against 5 μM Aβ_{42}-induced loss of cell viability in HT22 cells.

E. Change in cell number following treatment with 5 μM Aβ_{42} or 5 μg/mL petunidin alone, or Aβ_{42} + petunidin in combination for 24 h.

F. Change in gross cell morphology (a microscopic image of crystal violet-stained cells) following treatment with 5 μM Aβ_{42} or Aβ_{42} + petunidin in combination for 24 h. Red arrows indicate less healthy cells with abnormal morphology. Scale bar = 50 μm.

G. Quantification of crystal violet-stained cells following the same treatment as in **E.**

Bars represent mean ± S.D. (n = 3–5). $^*P < 0.05$ *vs.* control group; $^\#P < 0.05$ *vs.* Aβ_{42}-treated group.

Figure S2. Effect of petunidin on intracellular A*β* accumulation in Aβ_{42}-treated HT22 cells.

A. Change in A*β* accumulation following treatment with 5 μM Aβ_{42} or Aβ_{42} + petunidin in combination or Aβ_{42} + NAC in combination for 24 h. A representative image from each group is shown (yellow scale bar = 10 μm).

B. Quantitative analysis of the image shown in **A** (using the Image J software) reflecting the green/blue fluorescence ratio, which represents the relative index for amyloid load.

Bars represent mean ± S.D. (n = 3). $^*P < 0.05$ *vs.* control group; $^\#P < 0.05$ *vs.* Aβ_{42}-treated group.

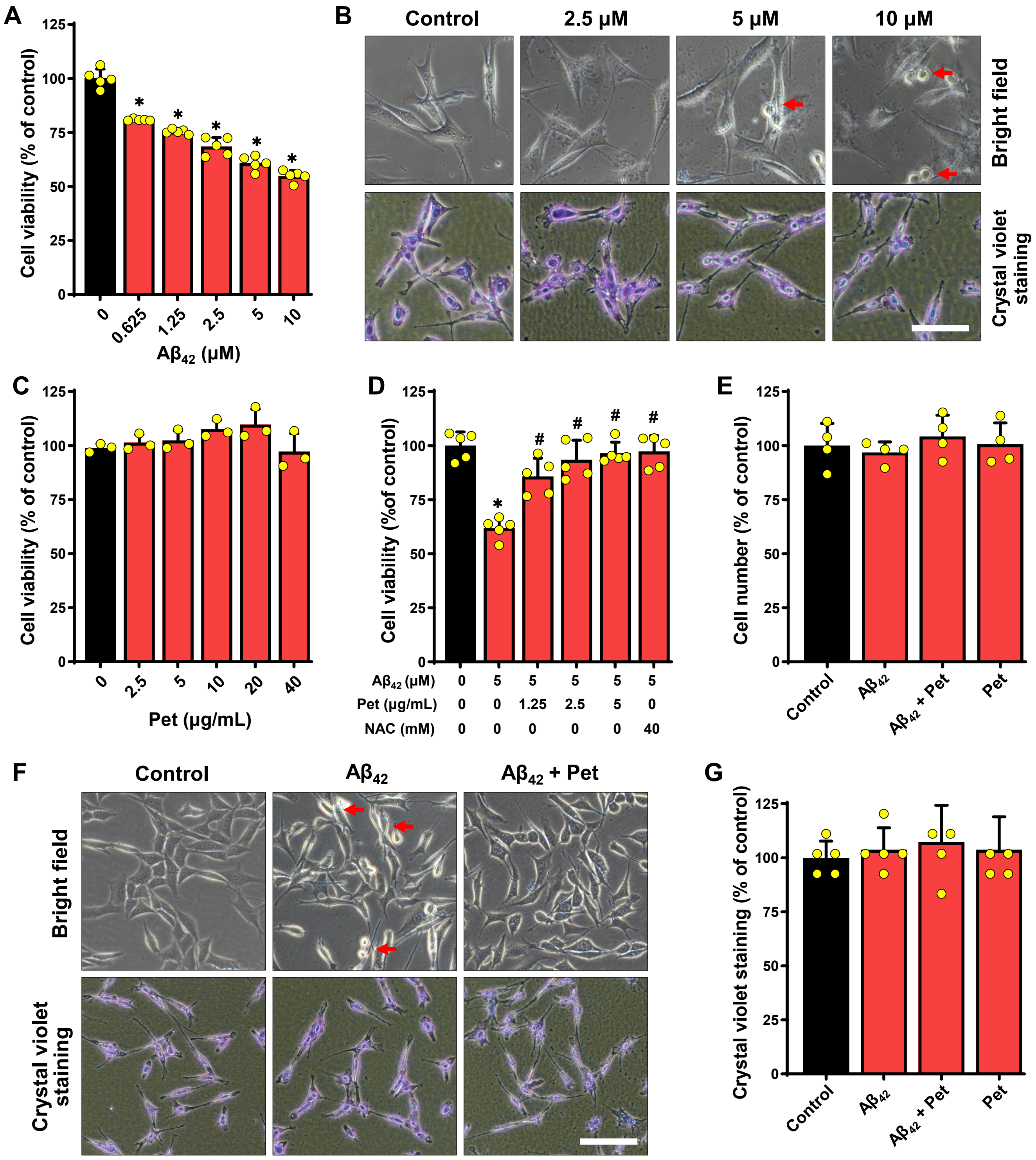
A
Cell viability (% of control)
Aβ42 (μM)
0
0.625
1.25
2.5
5
10
B
Control
2.5 μM
5 μM
10 μM
Bright field
Crystal violet staining
C
Cell viability (% of control)
Pet (μg/mL)
D
Cell viability (%of control)
Aβ42 (μM) 0 5 5 5 5 5
Pet (μg/mL) 0 0 1.25 2.5 5 0
NAC (mM) 0 0 0 0 0 40
E
Cell number (% of control)
Control
Aβ42
Aβ42 + Pet
Pet
F
Control
Aβ42
Aβ42 + Pet
Bright field
Crystal violet staining
G
Crystal violet staining (% of control)
Control
Aβ42
Aβ42 + Pet
Pet

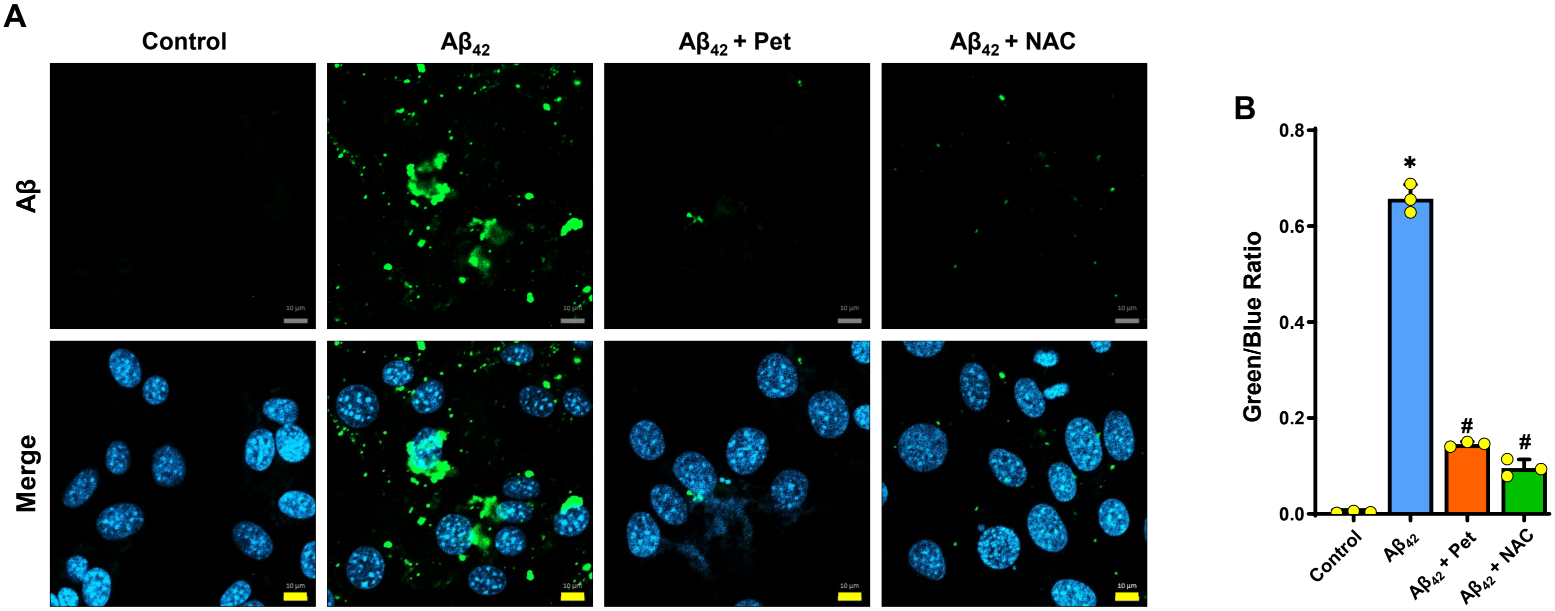

A
Control
Aβ42
Aβ42 + Pet
Aβ42 + NAC
Aβ
Merge
B
Green/Blue Ratio
0.8
0.6
0.4
0.2
0.0
*
#
#
Control
Aβ42
Aβ42 + Pet
Aβ42 + NAC
